# Bacteremia Associated With a Toothpick Lodged in the Duodenal Wall

**DOI:** 10.7759/cureus.33888

**Published:** 2023-01-17

**Authors:** Shahbaz Khan, Shakir Ullah, Masroor Anwar, Ravi Athwani, Khalid Nawab

**Affiliations:** 1 Internal Medicine, Ayub Medical College, Abbottabad, PAK; 2 Internal Medicine, Charles R. Drew University, Los Angeles, USA; 3 Internal Medicine, Khyber Teaching Hospital, Peshawar, PAK; 4 Internal Medicine, Penn State Holy Spirit Hospital, Camp Hill, USA; 5 Internal Medicine, Penn State Holy Spirit Medical Center, Camp Hill, USA

**Keywords:** foreign body ingestion, duodenitis, abdominal pain, toothpick, bacteremia

## Abstract

Toothpick ingestion and perforation of the gastrointestinal tract, although a very rare phenomenon, carries a very high mortality risk. Most cases of toothpick ingestion remain unnoticed until very late. The symptoms are often vague, with the most common being abdominal pain. Any obscure case of abdominal pain with bacteremia must be investigated for foreign body ingestion and perforation. A CT scan is the best initial diagnostic modality for toothpick perforation but has low sensitivity with laparoscopy, and endoscopy is the preferred diagnostic tool. No single bacterium is involved in bacteremia due to toothpick ingestion. Surgical or endoscopic removal of the impacted toothpick along with prompt antibiotic therapy leads to excellent outcomes. If left untreated or in case of a late diagnosis, it may lead to life-threatening consequences. Here, we present a case of a 44-year-old man who had ingested a toothpick that pierced through and was lodged in the duodenal wall, leading to bacteremia. The patient was successfully treated and discharged.

## Introduction

Foreign body ingestion is a relatively common phenomenon. About 90% of ingested foreign bodies pass uncomplicated through the body. But some sharp foreign bodies can get impacted in the gastrointestinal (GI) tract and cause serious complications [1]. Toothpick ingestion, specifically, is a relatively rare phenomenon and accounts for 8-9% of all ingested foreign bodies [2]. Its non-specific symptoms, inability to recall toothpick ingestion in up to 50% of cases [3], and its radiolucent nature makes it difficult to diagnose on routine investigations [4]. Ingestion of a toothpick carries a high mortality risk and may result in GI tract perforation as well as other serious consequences [5], with one study reporting a mortality rate of up to 18% [6]. We present a case report of a 44-year-old male who accidentally ingested a toothpick.

## Case presentation

A 44-year-old man presented to the emergency department with abdominal pain, subjective chills, and fever. The pain was in the mid-abdomen, non-radiating, sharp in nature, 3/10 in severity, tender to palpation, exacerbated with movement, and relieved with rest. The patient reported no chest pain, shortness of breath, or palpitations. The patient had no other significant past medical history. At the time of presentation, the patient had a temperature of 102 F. He was also tachycardic with a heart rate of 112/min. Blood pressure was normal at 112/73 mmHg and was saturating around 95% on room air.

His blood work revealed some leukocytosis, with a white blood cell count of 13 K/uL (reference range: 4.00 - 10.80 K/uL). Hemoglobin and platelet counts were in the normal range. The only other abnormal result was procalcitonin, which was elevated at 13.80 ng/mL (reference range: <0.1 ng/mL).

On diagnostic imaging, a CT scan of the abdomen with intravenous but without oral contrast showed inflammation of the duodenum suggesting duodenitis. No foreign body was noted on the CT scan.

**Figure 1:**
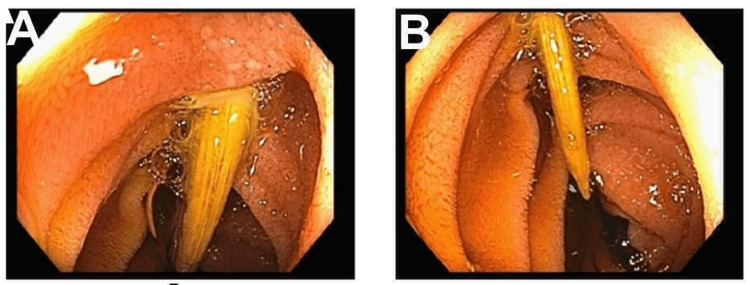
Third portion of the duodenum showing the toothpick lodged in the duodenal wall

The patient was admitted to the hospitalist service with the impression of sepsis, possibly secondary to duodenitis. He was started on IV pantoprazole as well as an empiric intravenous antibiotic (piperacillin/tazobactam 3.375 g every 8 hours). The patient continued to be febrile, eventually developing hypotension, requiring transfer to the intensive care unit and vasopressor support. Blood cultures were growing Streptococcus anginosus. Given persistent fever and bacteremia, there were concerns for endocarditis. The patient underwent a trans-esophageal echocardiogram that did not show any vegetation. The source of the infection remained unclear. However, on further questioning, the patient reported that a week before presentation to the hospital, the patient swallowed a toothpick by accident while drinking water. Gastroenterology was not convinced about potential foreign body leading to bacteremia, however, given the unclear source of bacteremia, the patient eventually underwent esophagogastroduodenoscopy, which revealed a large toothpick lodged in the wall of the third portion of the duodenum as shown in Figure 1, which was successfully removed by endoscopy. After the removal of the toothpick, the patient’s status improved significantly clinically over the subsequent 24 hours with a resolution of the fever and repeat blood cultures not showing any growth. A central venous catheter was placed, and the patient was discharged home on IV ceftriaxone 2 g for two more weeks.

## Discussion

The symptoms of toothpick ingestion can be highly nonspecific and can range from just abdominal pain, fever, and nausea to life-threatening conditions like bacteremia and shock. The literature review has found highly varying symptoms from hematochezia to abscess formation, and fistula formation to aortoenteric fistula. In our case, the majority of patients (82%) reported just abdominal pain [3].

The symptoms of toothpick ingestion depend greatly on the site of impaction. In our case, it was the third part of the duodenum. The toothpick had not perforated the gut but it is reported that about 80% of cases of toothpick ingestion result in perforation of the gastrointestinal tract [7]. The most common sites of impaction are reported to be the esophagus (2%), stomach (20%), duodenum (23%), small intestine (18%), and large intestine (37%) [8], but the literature review also reveals reports where the toothpick was found in the liver, pancreas, kidney, heart, bladder, ureter, and vasculature [4,9,10]. It can also lead to the formation of a fistula and pseudo-diverticulum as well as an abscess in different viscera [3,11]. These non-specific symptoms present a nightmare scenario for physicians and often lead to misdiagnosis. Unlike the majority of cases, our patient was able to recall the toothpick ingestion though it was not reported on initial evaluation. Therefore, foreign body ingestion like that of a toothpick may not be on the physician’s differentials, a thorough history may likely point to it.

No single bacterium has been implicated in cases of bacteremia due to toothpick ingestion. In our case, the cultures returned positive for Streptococcus anginosus but a review of the literature found many other bacteria like Streptococcus mitis [4], Enterococcus faecium, Enterobacter cloacae [3], Staphylococcus aureus [12], Escherichia coli [2], and Klebsiella pneumonia [13].^ ^So it is prudent to say that in patients presenting with gram-positive or gram-negative bacteremia with no obvious source, one should consider foreign body ingestion as a cause of obscure bacteremia [12].

Several imaging modalities can be used to find the presence of toothpick ingestion and perforation, including X-rays, CT scans, MRIs, and ultrasounds. Early diagnosis and retrieval of the toothpick are very important for reducing the mortality and morbidity associated with its ingestion and perforation [14]. Imaging studies though vary in sensitivity in detecting toothpicks and often prove to be inadequate [15]. Thus they can easily be overlooked [16] as was in our case, where the CT scan only showed the presence of inflammation and not a foreign body.

Definitive diagnosis can be made by explorative laparotomy [17],^ ^but it is rather an invasive procedure and assessment of objects lodged in the upper and lower GI tract. Gastroduodenoscopy and colonoscopy are the preferred choices because of their ability to visualize the area involved [18].

We used flexible endoscopy for the diagnosis and removal of the toothpick. Flexible endoscopy is the treatment of choice for upper GI toothpick removal depending on the location [12].

## Conclusions

Toothpick ingestion is often overlooked and can be dangerous leading to perforation of the GI tract and life-threatening bacteremia. Toothpick ingestion and perforation mostly present as abdominal pain, fever, and septicemia, thus it is difficult to diagnose without a history of ingestion. No single bacterium is associated with toothpicks or other foreign bodies lodged in the GI tract. A CT scan may show the presence of a toothpick but eventually, endoscopy and even laparoscopy may be required to remove the toothpick. If left undiagnosed and untreated, toothpick perforation can lead to high mortality and morbidity.
